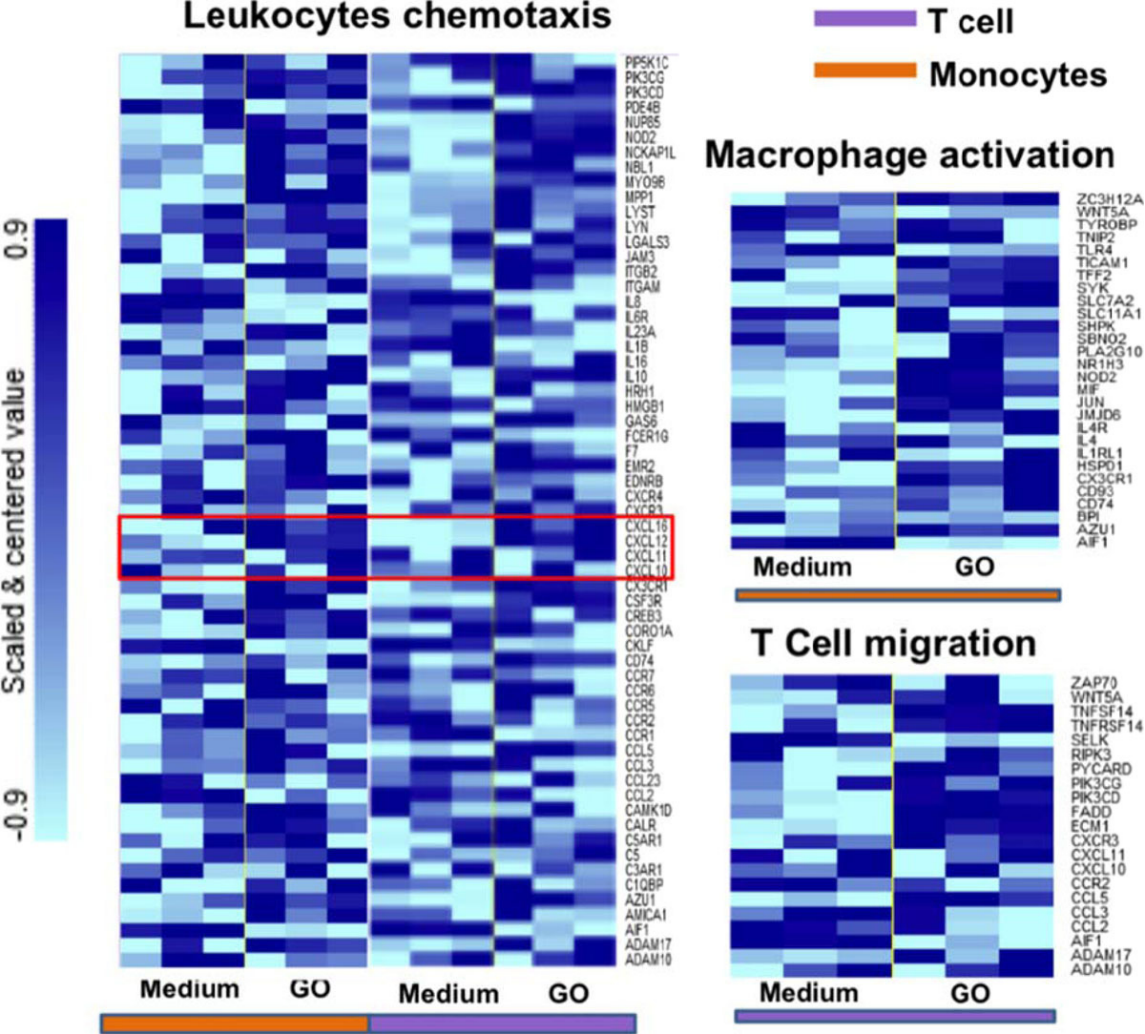# Molecular impact of graphene oxide with different shape dimension on human immune cells

**DOI:** 10.1186/2051-1426-3-S2-P217

**Published:** 2015-11-04

**Authors:** Marco Orecchioni, Dhifaf Jasim, Mario Pescatori, Francesco Sgarrella, Davide Bedognetti, Alberto Bianco, Kostas Kostarelos, Lucia Delogu

**Affiliations:** 1 University of Sassari, Sassari, Italy; 2 Faculty of Medical & Human Sciences, University of Manchester, Manchester, UK; 3 Sidra Medical and Research Center, Doha, Qatar; 4 CNRS, Institute de Biologie Moléculaire et Cellulaire, Strasbourg, France

## 

In the last few years, there has been enormous interest in graphene oxide (GO) for its wide variety of applications[1]. However, for any medical application, the immune system-impact of GO still remain to be fully understood. Moreover, the modulation of immune cells mediated by nanomaterials could be interesting also in immunotheraphy applications[2]. Indeed, nanomaterials and more in general nanotechnology can enhance the efficacy of immunostimulatory small molecules and biologics by altering their co-localization, biodistribution, and release kinetics[3].

**Figure 1:**
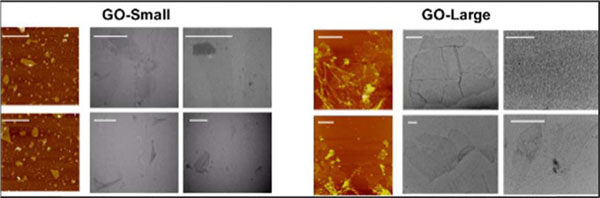


**Figure 2:**
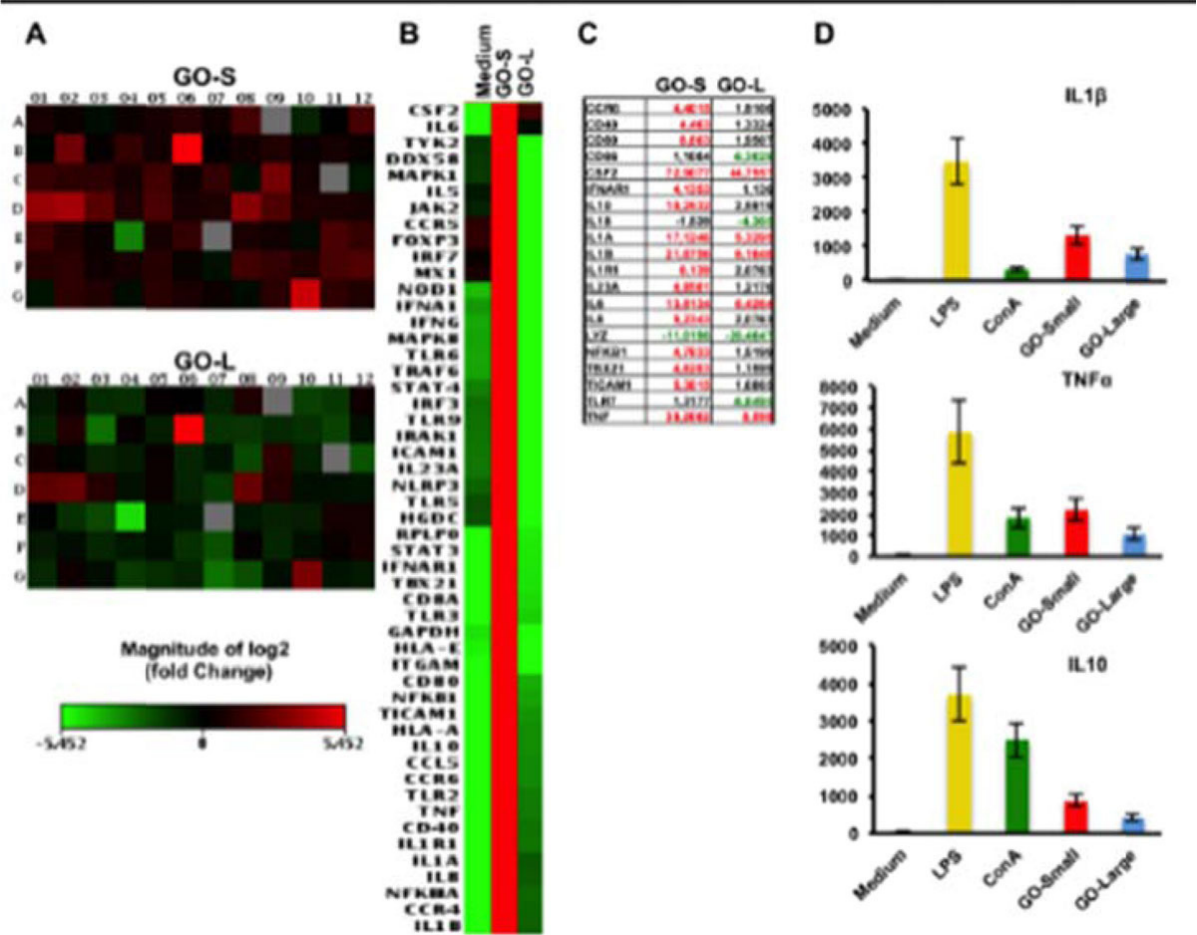


Following these aims we focused on the molecular effects of two GOs, different for lateral size dimensions, on human peripheral blood mononuclear cells (PBMCs). GOs were fully characterized then, we performed a wide range of standard assays looking at cell viability, cell activation and multiple cytokines secretion. We characterized the molecular impact of GOs on 84 genes immune-response-related. Additionally, a whole genome analysis was conducted on T cells and monocytes as representative of the innate and adaptive immune responses. In Figure 1 TEM and AFM characterization of GO-Small (140 nm) and GO-Large (4mm). We did not detect any toxicity in GO PBMCs treated samples. The 84 gene expression analysis evidenced a clear dimension-dependent impact of GOs on cell activation (Figure 2). In particular, the GO-Small modulated 16 genes (Fold Regulation >4) compared to only 5 of GO-Large (in red in Figure 2 C). Action confirmed also by cytokine analysis (Figure 2 D). These evidences were also confirmed by microarray analysis on T and monocytes cell lines. GO-Small impact the immune cell activation, underlined by the over expression of many pathways such as leukocyte chemotaxis pathway (Figure 3), genes such as CXCL10 ligand pathway and CXCR3 receptor (Figure 3, red box). Moreover, we found a strong action on cell metabolism with a down-regulation on energetic pathways such as oxidative-phosphorylation pathway in both cell types (data not shown). Our work represents a comprehensive molecular-characterization of different sized GOs on immune cells giving crucial information for the chemical and physical design of graphene for biomedical applications i.e. as a new possible drug delivery systems and nanoimmunotherapy tools.

**Figure 3:**